# Mitochondrial markers predict recurrence, metastasis and tamoxifen-resistance in breast cancer patients: Early detection of treatment failure with companion diagnostics

**DOI:** 10.18632/oncotarget.19612

**Published:** 2017-07-27

**Authors:** Federica Sotgia, Marco Fiorillo, Michael P. Lisanti

**Affiliations:** ^1^ Translational Medicine, School of Environment & Life Sciences, University of Salford, Greater Manchester, United Kingdom; ^2^ The Department of Pharmacy, Health and Nutritional Sciences, The University of Calabria, Cosenza, Italy

**Keywords:** mitochondria, mitochondrial biogenesis, biomarkers, treatment failure, relapse

## Abstract

Here, we used a data-mining and informatics approach to discover new biomarkers of resistance to hormonal therapy in breast cancer. More specifically, we investigated whether nuclear-encoded genes associated with mitochondrial biogenesis can be used to predict tumor recurrence, distant metastasis and treatment failure in high-risk breast cancer patients. Overall, this strategy allowed us to directly provide *in silico* validation of the prognostic value of these mitochondrial components in large and clinically relevant patient populations, with >15 years of follow-up data. For this purpose, we employed a group of 145 ER(+) luminal A breast cancer patients, with lymph-node (LN) metastasis at diagnosis, that were treated with tamoxifen, but not any chemotherapy agents. Using this approach, we identified >60 new individual mitochondrial biomarkers that predicted treatment failure and tumor recurrence, with hazard-ratios (HR) of up to 4.17 (*p*=2.2e-07). These include mitochondrial chaperones (HSPD1, HSPA9), membrane proteins (VDAC2, TOMM70A) and anti-oxidants (SOD2), as well as 18 different mitochondrial ribosomal proteins (MRPs) and >20 distinct components of the OXPHOS complexes. In addition, we combined 4 mitochondrial proteins (HSPD1, UQCRB, MRPL15, COX17), to generate a compact mitochondrial gene signature, associated with a HR of 5.34 (*p*=1e-09). This signature also successfully predicted distant metastasis and was effective in larger groups of ER(+) (*N*=2,447), basal (*N*=540) and HER2(+) (*N*=193) breast cancers. It was also effective in all breast cancers (*N*=3,180), if considered together as a single group. Based on this analysis, we conclude that mitochondrial biogenesis should be considered as a new therapeutic target for overcoming tumor recurrence, distant metastasis and treatment failure in patients with breast cancer. In summary, we identified individual mitochondrial biomarkers and 2 compact mitochondrial gene signatures that can be used to predict tamoxifen-resistance and tumor recurrence, at their initial diagnosis, in patients with advanced breast cancer. In the long-term, these mitochondrial biomarkers could provide a new companion diagnostics platform to help clinicians to accurately predict the response to hormonal therapy in ER(+) breast cancer patients, facilitating more personalized and effective treatment. Similarly, these mitochondrial markers could be used as companion diagnostics, to determine which breast cancer patients would benefit most from clinical treatments with mitochondrially-targeted anti-cancer therapeutics. Finally, we also showed that these mitochondrial markers are superior when directly compared with conventional biomarkers, such as Ki67 and PCNA.

## INTRODUCTION

Treatment failure, due to drug resistance, still remains a major obstacle for more effective anti-cancer therapy and personalized medicine [1–9]. In estrogen-receptor-positive (ER(+)) breast cancer, approximately 40-to-50% of patients eventually develop tamoxifen-resistance [5–9]. Importantly, the five-year survival rate following tamoxifen-resistance is less than 20% [1–5]. Unfortunately, tamoxifen-resistance often manifests itself as tumor recurrence and/or distant metastasis. As such, resistance to endocrine therapy is a critical factor that still limits the efficacy of breast cancer treatment. Thus, better biomarkers and companion diagnostics are needed for the early detection of patients that will likely fail hormonal therapy [5–9].

Here, we set out to test the hypothesis that individual markers of mitochondrial biogenesis and OXPHOS may have prognostic value in the early identification of tamoxifen-resistant patients at diagnosis, up to 15 years before the onset of tumor recurrence and distant metastasis. For this purpose, we performed outcome analysis on > 400 nuclear mitochondrial gene transcripts.

Our results indicate that > 60 different mitochondrial markers can be used individually or in combination, as short signatures, to predict tumor recurrence in tamoxifen-treated breast cancer patients. As a consequence, we discuss the possibility that mitochondria should be therapeutically targeted, to overcome resistance to hormonal therapy, and to prevent tumor recurrence and distant metastasis. In accordance with this idea, metformin (a mitochondrial complex I inhibitor) has been previously shown to overcome tamoxifen-resistance in ER(+) cell culture models, which mimic the tumor microenvironment by the addition of stromal fibroblasts [9–11].

Interestingly, mitochondrial markers also showed prognostic value in different sub-groups of ER(−) breast cancer patients [12].

## RESULTS

### Establishing the prognostic value of conventional markers in the patient population

**Figure 1:**
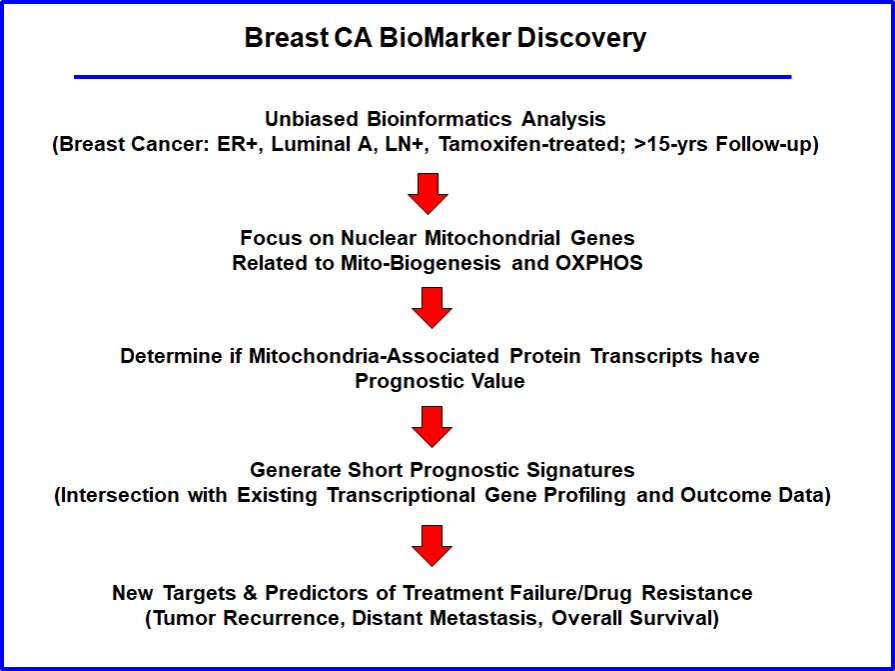
Flow-diagram illustrating our overall informatics approach to breast cancer biomarker discovery for predicting tamoxifen-resistance For this analysis, we chose to focus on ER(+) patients, luminal A sub-type, that were lymph-node positive (LN(+)) at diagnosis, who were treated with tamoxifen and followed over a period of nearly 200 months (> 15 years). In this context, we evaluated the prognostic value of mitochondrial markers for predicting tumor recurrence or distant metastasis (treatment failure), as well as overall survival, in this patient population.

To identify new potential biomarkers of tamoxifen-resistance, here we used publically available transcriptional profiling data from the tumors of breast cancer patients that were treated with tamoxifen, but did not receive any chemotherapy. For this purpose, we selected high-risk patients that were lymph-node positive at diagnosis, and we focused on the luminal A subtype, which represents the most common form of ER(+) breast cancers (*N* = 145 patients) (Figure 1).

**Figure 2:**
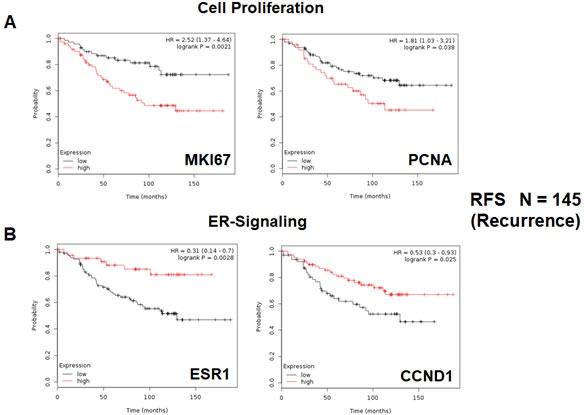
Conventional markers of proliferation and estrogen-receptor-alpha signaling predict clinical outcome in high-risk ER(+) breast cancer patients We assessed the predictive value of Ki67 and PCNA in *N* = 145 ER(+) breast cancer patients, luminal A sub-type, that were lymph-node positive (LN(+)) at diagnosis, who were treated with tamoxifen and followed over a period of nearly 200 months (> 15 years). **A.** Note that high transcript levels of Ki67 and PCNA are associated with increased levels of tumor recurrence, indicative of tamoxifen-resistance. Please note that the official gene name for the Ki67 protein is MKI67. **B.** Note that high transcript levels of estrogen-receptor (ESR1) and cyclin D1 expression (CCND1) are both associated with reduced tumor recurrence, showing increased efficacy of tamoxifen therapy. RFS, recurrence or relapse free survival is shown (a.k.a., tumor recurrence).

As proliferative markers are often used as the primary endpoint in clinical trials, we first assessed the prognostic value of Ki67 and PCNA, in this patient population. Table 1 and Figure 2A both show the prognostic value of these markers. The hazard-ratios for Ki67 and PCNA were 2.5 and 1.8, respectively, for relapse-free survival (RFS) (i.e., tumor recurrence).

**Table 1:** Prognostic value of known markers of proliferation

Gene Probe ID	Symbol	Hazard-Ratio	Log-Rank Test
212022_s_at	MKI67	2.52	0.002
217400_at	PCNA	1.81	0.04

Next, we assessed the behavior of markers of estrogen receptor signaling in these patients. It would be predicted that increased levels of such markers would be associated with a positive response to hormonal therapy. As predicted, Table 2 and Figure 2B show that estrogen receptor-alpha (ESR1) and cyclin D1/2 levels (CCND1/2) both effectively predict tamoxifen-sensitivity, as reflected by a reduction in tumor recurrence.

**Table 2:** Prognostic value of known markers of ER-Signaling

Gene Probe ID	Symbol	Hazard-Ratio	Log-Rank Test
205225_at	ESR1	0.31	0.003
208711_s_at	CCND1	0.53	0.025
200952_s_at	CCND2	0.50	0.03

**Figure 3:**
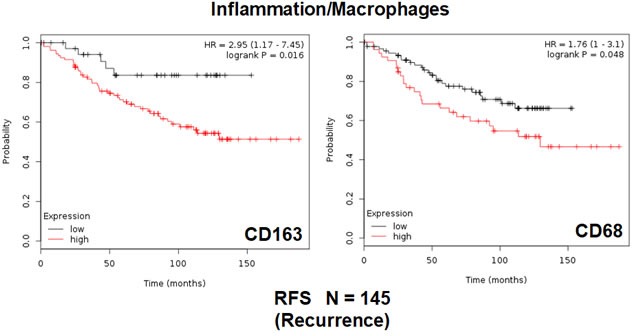
Conventional markers of macrophage-associated inflammation predict poor clinical outcome in high-risk ER(+) breast cancer patients Note that that high transcript levels of CD163 and CD68 are associated with increased levels of tumor recurrence and, therefore, tamoxifen-resistance.

Finally, we also assessed the prognostic value of two macrophage-specific markers of inflammation. Table 3 and Figure 3 show that CD68 and CD163 both effectively predict tumor recurrence, with hazard-ratios of 1.76 and 2.95, respectively.

**Table 3:** Prognostic value of markers of inflammation

Gene Probe ID	Symbol	Hazard-Ratio	Log-Rank Test
216233_at	CD163	2.95	0.02
215049_x_at	CD163	2.45	0.009
203645_s_at	CD163	2.34	0.003
203507_at	CD68	1.76	0.048
**Combined**		**2.31**	**0.003**

Thus, conventional markers of proliferation, estrogen signaling, and inflammation can all be used to predict tumor-recurrence and tamoxifen-resistance in LN(+) luminal A breast cancer patients.

### Prognostic value of individual markers of mitochondrial biogenesis

To test our hypothesis that increased mitochondrial biogenesis contributes towards tumor recurrence and tamoxifen-resistance, we next assessed the prognostic value of specific mitochondrial markers.

**Figure 4:**
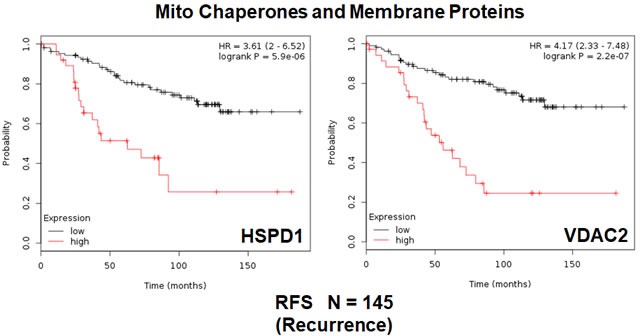
Mitochondrial chaperones and membrane proteins are associated with tumor recurrence in high-risk ER(+) breast cancer patients Note that that high transcript levels of HSPD1 and VDAC2 are associated with increased levels of tumor recurrence and resistance to hormonal therapy.

First, we examined the behavior of mitochondrial chaperones (HSPs) and mitochondrial membrane proteins (TIMM/TOMM/VDAC families). Table 4 and Figure 4 show that HSP60 (HSPD1) and VDAC2 have the best prognostic value with hazard-ratios of 3.6 and 4.2, respectively. Importantly, several members of the TIMM and TOMM gene families also had prognostic value (HR = 1.8-to-2.8). AKAP1 and IMMT also had significant value (HR = 1.8-to-2.2). Notably, the mitochondrial anti-oxidant SOD2 also showed significant prognostic value, with a hazard-ratio of 2.94 (*p* = 0.0001) (Table 4). Similar results were obtained with mitochondrial creatine kinase isoforms (HR = 2.0-to-2.2).

**Table 4:** Prognostic value of mitochondrial chaperones, membrane proteins, carriers, anti-oxidants and creatine kinase

Gene Probe ID	Symbol	Hazard-Ratio	Log-Rank Test
**Mito Chaperones**			
200807_s_at	HSPD1	3.61	5.9e-06
200806_s_at	HSPD1	2.30	0.006
200691_s_at	HSPA9	2.04	0.01
205565_s_at	FXN	1.83	0.038
221235_s_at	TRAP1	1.79	0.047
**Mito Membrane Proteins**			
211662_s_at	VDAC2	4.17	2.2e-07
210626_at	AKAP1	2.15	0.01
200955_at	IMMT	1.81	0.04
201519_at	TOMM70A	2.78	0.0003
201512_s_at	TOMM70A	2.15	0.01
203093_s_at	TIMM44	2.23	0.01
218188_s_at	TIMM13	2.23	0.02
201822_at	TIMM17A	2.01	0.01
215171_s_at	TIMM17A	1.85	0.04
203342_at	TIMM17B	1.78	0.04
**Mito Carrier Family**			
217961_at	SLC25A38	2.77	0.0003
210010_s_at	SLC25A1	2.38	0.002
200657_at	SLC25A5	2.04	0.01
221020_s_at	SLC25A32	1.98	0.02
**Mito Anti-Oxidants**			
215223_s_at	SOD2	2.94	0.0001
215078_at	SOD2	2.81	0.008
**Mito Creatine Kinase**			
205295_at	CKMT2	2.18	0.04
202712_s_at	CKMT1A	2.03	0.02

Next, we examined the prognostic value of all the known mitochondrial ribosomal proteins (MRPs), which contribute to the protein translation of key members of the OXPHOS-related complexes, and are essential for mitochondrial biogenesis (summarized in Table 5).

**Table 5:** Prognostic value of mitochondrial Ribosomal proteins

Gene Probe ID	Symbol	Hazard-Ratio	Log-Rank Test
**Large Ribosomal Subunit**			
218027_at	MRPL15	3.28	1.6e-05
217907_at	MRPL18	2.91	0.0001
219244_s_at	MRPL46	2.89	0.02
218270_at	MRPL24	2.38	0.002
218049_s_at	MRPL13	2.14	0.01
218281_at	MRPL48	2.11	0.01
208787_at	MRPL3	2.07	0.03
213897_s_at	MRPL23	2.02	0.04
218105_s_at	MRPL4	1.99	0.02
222216_s_at	MRPL17	1.97	0.02
217919_s_at	MRPL42	1.88	0.05
218202_x_at	MRPL44	1.78	0.04
**Small Ribosomal Subunit**			
204330_s_at	MRPS12	2.35	0.03
211595_s_at	MRPS11	2.26	0.01
219819_s_at	MRPS28	1.88	0.03
217919_s_at	MRPL42	1.88	0.05
219220_x_at	MRPS22	1.85	0.04
218654_s_at	MRPS33	1.84	0.04

Twelve different components of the large subunit (MRPLs) showed significant prognostic value, with hazard-ratios between 1.8 and 3.3. Most notably, MRPL15 had the best prognostic value (HR = 3.3; *p* = 1.6e-05). Similarly, six different components of the small subunit (MRPSs) showed significant prognostic value, with hazard-ratios between 1.8 and 2.35.

**Figure 5:**
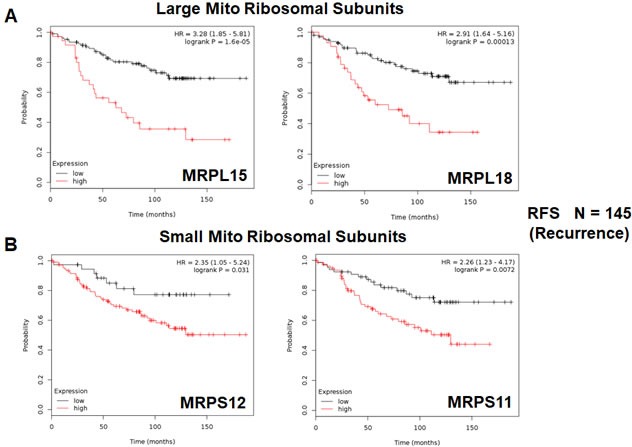
Mitochondrial ribosomal proteins (MRPs) are associated with tumor recurrence in high-risk ER(+) breast cancer patients **A.** Note that high transcript levels of MRPL15 and MRPL18 predict increased tumor recurrence and tamoxifen-resistance. **B.** Similarly, high transcript levels of MRPS12 and MRPS11 predict increased tumor recurrence and tamoxifen-resistance.

Thus, 18 different MRPs all predicted tumor recurrence. Kaplan-Meier curves for representative examples are shown in Figure 5, panels A & B.

We also assessed the prognostic value of members of the OXPHOS complexes I-V. These results are summarized in Table 6. Remarkably, greater than 20 different members of the OXPHOS complexes showed hazard-ratios between 1.9 and 3.4. UQCRB (complex III) had the best prognostic value (HR = 3.42; *p* = 1.9e-05). Similarly, COX17 (complex IV) showed significant prognostic value (HR = 2.99; *p* = 7.6e-05).

**Table 6:** Prognostic value of mitochondrial OXPHOS complexes

Gene Probe ID	Symbol	Hazard-Ratio	Log-Rank Test
**Complex I**			
218160_at	NDUFA8	2.45	0.002
202000_at	NDUFA6	2.41	0.002
202001_s_at	NDUFA6	2.23	0.006
203039_s_at	NDUFS1	2.40	0.003
201740_at	NDUFS3	2.17	0.006
203613_s_at	NDUFB6	1.99	0.02
208714_at	NDUFV1	1.96	0.03
203606_at	NDUFS6	1.92	0.04
202298_at	NDUFA1	1.89	0.03
**Complex III**			
209065_at	UQCRB	3.42	1.9e-05
209066_x_at	UQCRB	2.12	0.01
205849_s_at	UQCR6	2.53	0.002
201066_at	UQCR4	1.96	0.02
212600_s_at	UQCRC2	1.92	0.04
**Complex IV**			
203880_at	COX17	2.99	7.6e-05
213735_s_at	COX5B	2.51	0.001
202343_x_at	COX5B	2.10	0.01
211025_x_at	COX5B	2.08	0.01
202698_x_at	COX4I1	2.36	0.02
200925_at	COX6A1	2.14	0.01
218057_x_at	COX4NB	1.99	0.04
217249_x_at	COX7A2	1.90	0.03
**Complex V**			
202325_s_at	ATP5J	2.65	0.01
202961_s_at	ATP5J2	2.44	0.035
213366_x_at	ATP5C1	2.19	0.01
208870_x_at	ATP5C1	2.08	0.01
205711_x_at	ATP5C1	2.00	0.02
217848_s_at	PPA1	2.07	0.01
221677_s_at	ATP5O	2.03	0.02
217801_at	ATP5E	1.99	0.02
207508_at	ATP5G3	1.93	0.02

**Figure 6:**
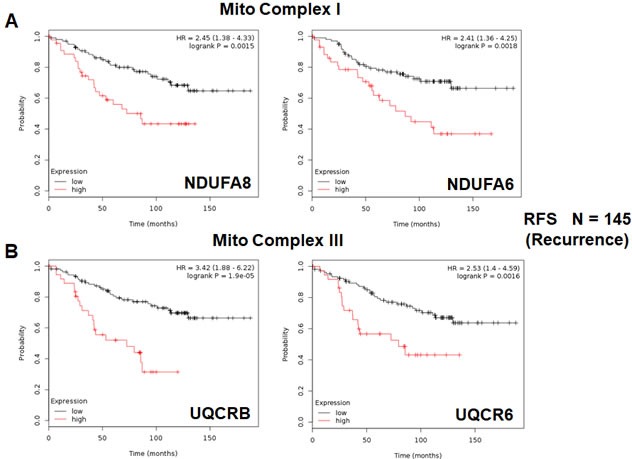
Mitochondrial complex I and complex III proteins are associated with tumor recurrence in high-risk ER(+) breast cancer patients **A.** Note that high levels of NDUFA8 and NDUFA6 predict increased tumor recurrence and tamoxifen-resistance. **B.** Similarly, high levels of UQCRB and UQCR6 predict increased tumor recurrence and tamoxifen-resistance.

**Figure 7:**
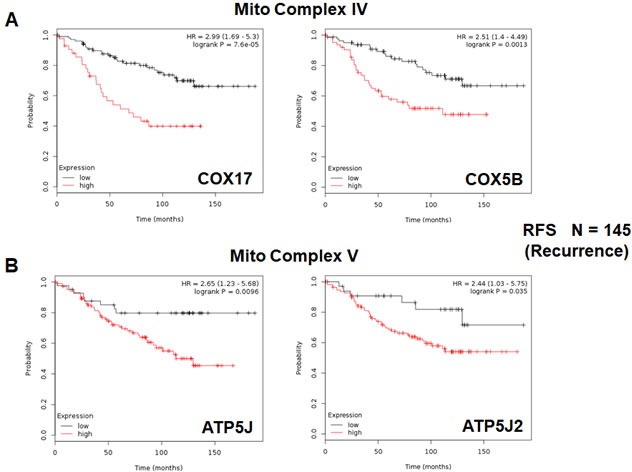
Mitochondrial complex IV and complex V proteins are associated with tumor recurrence in high-risk ER(+) breast cancer patients **A.** Note that high levels of COX17 and COX5B predict increased tumor recurrence and tamoxifen-resistance. **B.** Similarly, high levels of ATP5J and ATP5J2 predict increased tumor recurrence and tamoxifen-resistance.

Kaplan-Meier curves for members of complex I and III are shown in Figure 6A & 6B, while results with members of complex IV and V are also shown in Figure 7A & 7B.

### Two new mitochondrial gene signatures for predicting tumor recurrence, distant metastasis and tamoxifen-resistance

**Figure 8:**
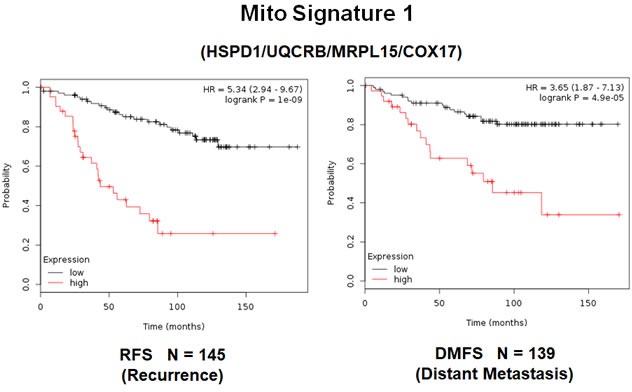
A short mitochondrial signature (Mito-Signature-1) predicts poor clinical outcome in high-risk ER(+) breast cancer patients Note that this short 4-gene signature (HSPD1/UQCRB/MRPL15/COX17) effectively predicts tumor recurrence and distant metastasis in LN(+) luminal A patients treated with tamoxifen therapy, indicative of treatment failure and tamoxifen-resistance.

**Figure 9:**
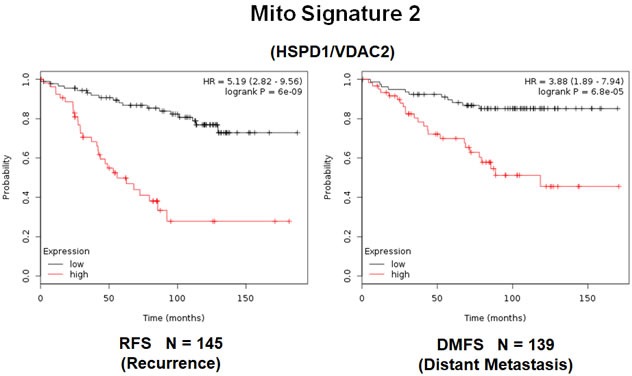
A short mitochondrial signature (Mito-Signature-2) predicts poor clinical outcome in high-risk ER(+) breast cancer patients Note that this short 2-gene signature (HSPD1/VDAC2) effectively predicts tumor recurrence and distant metastasis in LN(+) luminal A patients treated with tamoxifen therapy, indicative of treatment failure and tamoxifen-resistance.

In order to increase the prognostic power of these individual mitochondrial biomarkers, we next selected the most promising ones and used them to create two new mitochondrial gene signatures. Mito-Signature-1 contains 4 genes (HSPD1, UQCRB, MRPL15, COX17), while Mito-Signature-2 consists of only 2 genes (HSPD1, VDAC2) (See Tables 7 & 8). K-M curves for these two signatures are shown in Figures 8 and 9.

**Table 7:** Mito-signature 1 for predicting treatment failure

Gene Probe ID	Symbol	Hazard-Ratio	Log-Rank Test
200807_s_at	HSPD1	3.61	5.9e-06
209065_at	UQCRB	3.42	1.9e-05
218027_at	MRPL15	3.28	1.6e-05
203880_at	COX17	2.99	7.6e-05
**Combined**		**5.34**	**1e-09**

**Table 8:** Mito-signature 2 for predicting treatment failure

Gene Probe ID	Symbol	Hazard-Ratio	Log-Rank Test
211662_s_at	VDAC2	4.17	2.2e-07
200807_s_at	HSPD1	3.61	5.9e-06
**Combined**		**5.19**	**6e-09**

Importantly, Mito-Signature-1 yielded a significantly improved hazard-ratio for tumor recurrence of 5.34 (*p* = 1e-09). It was also highly predictive for distant metastasis, in the same group of patients (HR = 3.65; *p* = 4.9e-05).

Similarly, Mito-Signature-2 showed a hazard-ratio for tumor recurrence of 5.2 (*p* = 6e-09). Mito-Signature-2 was also highly predictive for distant metastasis (HR = 3.88; *p* = 6.8e-05).

Thus, both mitochondrial signatures were a significant improvement over individual mitochondrial biomarkers, as well as Ki67, PCNA, ESR1, CCND1/2 and CD68/CD163 (compare with Figures 2 & 3).

### Two short mitochondrial gene signatures can effectively predict tumor recurrence in larger ER(+) patient populations that received hormonal therapy, as well as in ER(−) patients, and all breast cancers, considered as a single group

We also examined the prognostic value of these two mitochondrial gene signatures in a larger group of ER(+) patients (*N* = 698), that received hormonal therapy, but not chemotherapy. This group of patients was not segregated into luminal A and luminal B subtypes.

**Figure 10:**
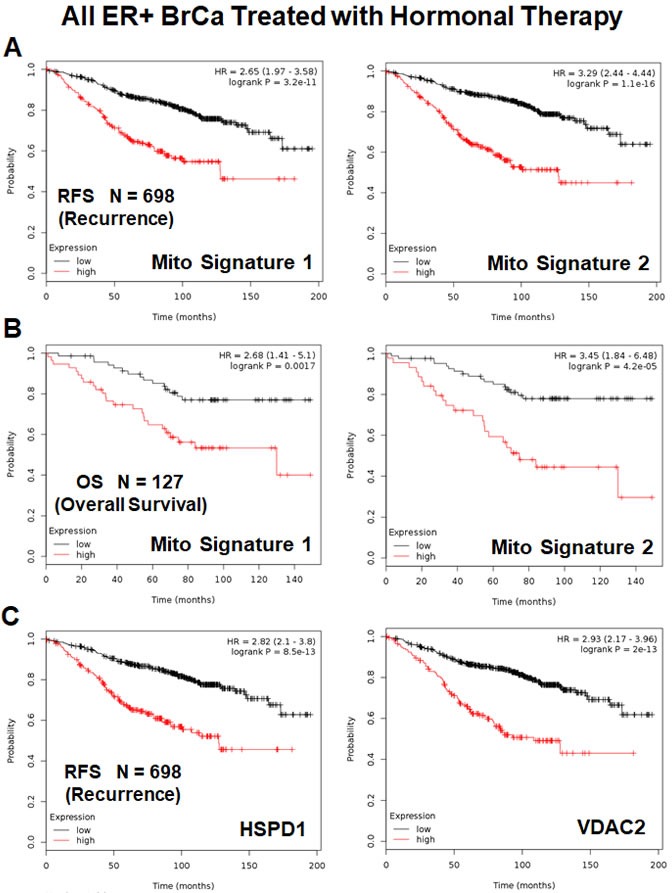
Mitochondrial signatures 1 and 2 both have predictive value in a larger group of ER(+) breast cancer patients, who were treated with hormonal therapy These patients were not sub-divided into luminal A/B subgroups and were not sub-divided by lymph-node status. **A.** K-M analysis with Mito-Signatures 1 & 2, showing tumor recurrence. *N* = 698 patients. **B.** K-M analysis with Mito-Signatures 1 & 2, showing overall survival. *N* = 127 patients. **C.** K-M analysis with individual markers (HSPD1 and VDAC2) is also shown for comparison. *N* = 698 patients.

Figure 10A shows the results of this K-M analysis for relapse-free survival: Mito-Signature-1 (HR = 2.65; *p* = 3.2e-11) and Mitosignature-2 (HR = 3.3; *p* = 1.1e-16). Similar results were also obtained for overall survival (Figure 10B).

**Figure 11:**
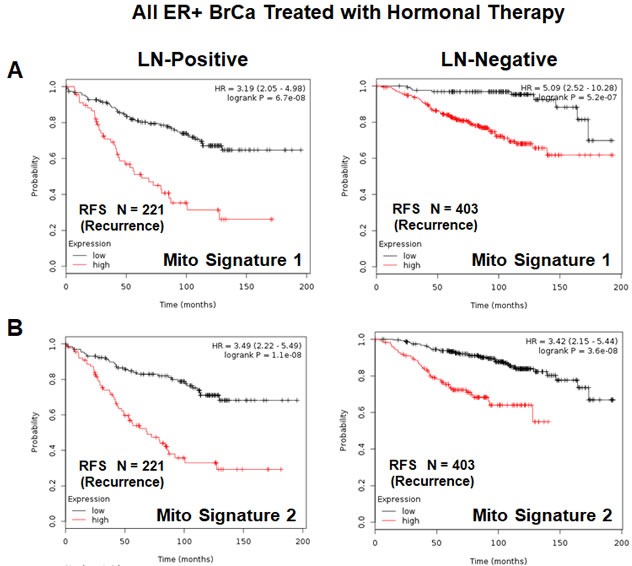
Mitochondrial signatures 1 and 2 both have predictive value in a larger group of ER(+) breast cancer patients, who were treated with hormonal therapy These patients were not sub-divided into luminal A/B subgroups, but were sub-divided by lymph-node status (LN(+) *versus* LN(−)). **A.** K-M analysis with Mito-Signature-1 is shown for both groups: LN(+) (*N* = 221 patients) and LN(−) (*N* = 403 patients). **B.** K-M analysis with Mito-Signature-2 is shown for both groups: LN(+) (*N* = 221 patients) and LN(−) (*N* = 403 patients).

**Figure 12:**
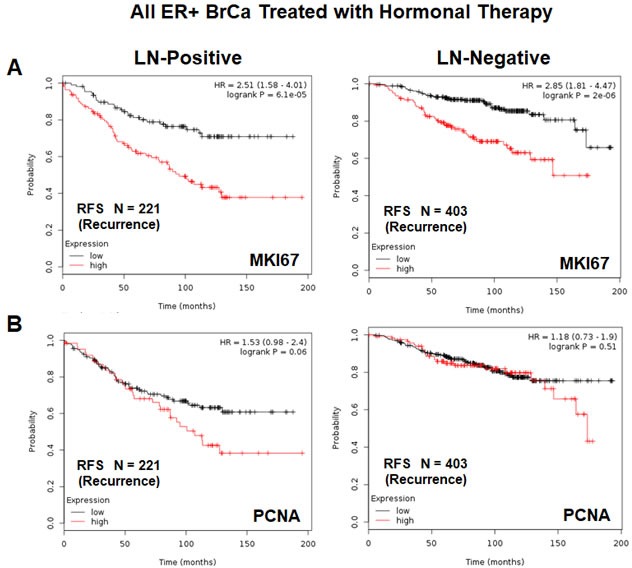
K-M analysis with conventional proliferative markers, in the same patient population, is shown for comparison Note that Mito Signature 1 & 2 show better predictive value than both proliferative markers, namely KI67 and PCNA. **A.** K-M analysis with KI67 is shown for both groups: LN(+) (*N* = 221 patients) and LN(−) (*N* = 403 patients). **B.** K-M analysis with PCNA is shown for both groups: LN(+) (*N* = 221 patients) and LN(−) (*N* = 403 patients).

Both of these mitochondrial signatures were also effective if the ER(+) patient population was divided into LN(+) and LN(−) groups (Figure 11A & 11B). In addition, both of these mitochondrial signatures were clearly superior to Ki67 and PCNA in this larger ER(+) patient population. However, Ki67 still showed prognostic value (Figure 12A), while PCNA had no prognostic value (Figure 12B).

Finally, we assessed the behavior of Mito-Signature-1 in even larger and more varied patient populations, where the therapy was not restricted to tamoxifen.

Supplementary Figure 1 shows that Mito-Signature-1 was also effective in ER(+) (*N* = 2,447), ER-/basal (*N* = 540), ER-/HER2(+) (*N* = 193), as well as in all breast cancer subtypes combined (*N* = 3,180). Similarly, Mito-Signature-1 was still statistically effective in both luminal A (*N* = 438 + 813) and luminal B (*N* = 907) patient populations (Supplementary Figure 2). Similarly, comparable results were obtained with Mito-Signature-2 (data not shown).

Thus, these mitochondrial-based gene signatures may represent important new prognostic tools for predicting patient outcomes, in a wide variety of different breast cancer patients, but especially in ER(+) patients treated with hormonal therapies.

## DISCUSSION

### Early detection of tamoxifen-resistance with mitochondrial markers: prevention of tumor recurrence and distant metastasis?

Here, we show that mitochondrial markers effectively predict tumor recurrence, distant metastasis and tamoxifen-resistance in high-risk ER(+) breast cancer patients. Importantly, these mitochondrial markers could now be used to identify high-risk ER(+) breast cancer patients at diagnosis, up to 15 years in advance, before they undergo tumor recurrence and metastasis. These results also suggest that mitochondria should be therapeutically-targeted in epithelial cancer cells to overcome tamoxifen-resistance and prevent the failure of hormonal therapy.

Consistent with this hypothesis, we have previously shown that treatment with metformin (a mitochondrial complex I inhibitor) is indeed sufficient to reverse tamoxifen-resistance in fibroblast-MCF7 co-cultures [10, 11]. Thus, targeting mitochondrial biogenesis and OXPHOS in ER(+) epithelial breast cancer cells may be a new therapeutic strategy for preventing or reversing tamoxifen-resistance in breast cancer patients.

Interestingly, these mitochondrial markers also showed predictive value in ER(−) breast cancer patients, both basal and HER2(+), suggesting that anti-mitochondrial therapy could be used as a more general anti-cancer strategy, against several different breast cancer sub-types.

**Figure 13:**
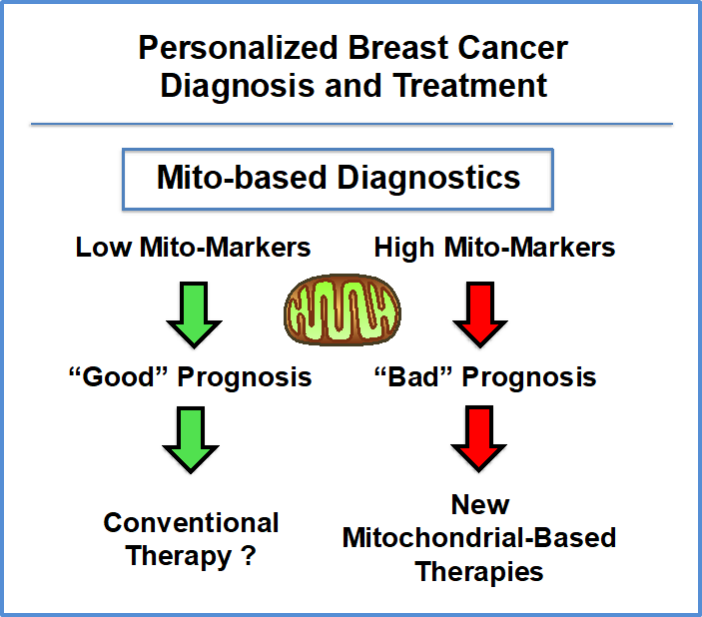
Mitochondrial-based companion diagnostics for personalized cancer therapy In this flow-diagram, mitochondrial-based diagnostics would be used to separate breast cancer patients into high-risk and low-risk groups. Then, patients with high levels of mitochondrial markers in their primary tumor (“bad prognosis”) would be treated with mitochondrial-based therapies (such as Metformin), as an add-on to the standard of care, to prevent tumor recurrence, distant metastasis and tamoxifen-resistance.

A schematic diagram summarizing this new mito-based approach is presented in Figure 13. In this workflow, high-risk patients are first identified at diagnosis by the high expression of mitochondrial markers in their primary breast tumors. Then, these patients would be treated with mitochondrial-based therapeutics (e.g., metformin or another FDA-approved drug; in combination with the standard of care), to help prevent tumor recurrence and distant metastasis. Alternatively, novel mitochondrial-based chemo-therapeutics could be developed against a variety of metabolic enzymes or structural proteins, to specifically target aggressive cancer cells with increased mitochondrial function.

### Evidence that mitochondrial power drives tamoxifen-resistance and cancer stem cell propagation

Consistent with the above hypothesis, we recently showed that tamoxifen-resistant MCF7 cells (TAMR) are characterized by a metabolic phenotype, consisting of i) increased mitochondrial biogenesis, ii) increased ATP production and iii) reduced glutathione levels [13]. Thus, inhibition of mitochondrial function may be a new therapeutic strategy for overcoming tamoxifen-resistance in breast cancer patients. These findings could have important translational significance for the prevention of tumor recurrence in ER(+) breast cancers, which is due to an endocrine resistance phenotype [13]. Importantly, mitochondrial proteins may represent i) new prognostic biomarkers, ii) novel therapeutic targets and iii) companion diagnostics, for predicting and overcoming tamoxifen-resistance in different subsets of ER(+) breast cancer patients.

Similarly, based on high-resolution proteomics analysis, we have also proposed that mitochondrial biogenesis is an important driver of the cancer stem cell (CSC) phenotype [14, 15]. A key correlate of this assertion is that high mitochondrial mass is a metabolic biomarker for CSCs. To directly test this idea experimentally, we used a fluorescent dye, known as MitoTracker, to detect and quantitate mitochondrial mass in ER(+) breast cancer cells (MCF7) [16]. Using this approach, we purified the Mito-high and the Mito-low cell populations by flow cytometry (FACS). Remarkably, the Mito-high cell population was clearly enriched in cells with the characteristics of CSCs. Virtually identical results were also obtained with MDA-MB-231 cells, an ER(−) cell line. Thus, the use of “metabolic fractionation”, employing mitochondrial-based probes and flow cytometry, could be a successful new approach to the functional purification of drug-resistant CSCs. In accordance with this hypothesis, Mito-high breast cancer cells were also resistant to DNA-damage induced by Paclitaxel [16]. Thus, mitochondrial mass and function are directly linked to i) the CSC phenotype and ii) chemotherapeutic drug resistance, as well as iii) resistance to anti-estrogen therapy [13–24]. As such, we conclude that the association we observed here of high levels of mitochondrial markers (mRNA species and/or protein products) with poor clinical outcome in breast cancer patients may functionally reflect the presence of drug-resistant CSCs, driving tumor recurrence, metastasis and treatment failure.

### Using mitochondrial markers as companion diagnostics for drug re-purposing, treatment stratification and new drug discovery

Several classes of FDA-approved antibiotics safely inhibit either mitochondrial biogenesis or OXPHOS as off-target “side-effects”. These include the tetracyclines (doxycycline), the erythromycins (azithromycin), pyrvinium pamoate, atovaquone, and bedaquiline, among others [13–24]. Therefore, the new mitochondrial biomarkers that we identified here could be used in combination with these FDA-approved drugs, as companion diagnostics. This would allow clinicians to select the right patient populations for new clinical trials aimed at drug re-purposing/re-positioning, for the prevention of tumor recurrence in ER(+) patients receiving anti-endocrine therapy.

Importantly, the novel mitochondrial biomarkers that we identified here may also be new therapeutic targets for future drug development aimed at combating the emergence of resistance to hormonal therapy. Based on our K-M analysis, the mitochondrial ribosome (a.k.a., mitoribosome) and its individual subunits would be attractive targets for intervention; in addition, mitochondrial chaperones, the OXPHOS complexes (I-IV) and the mitochondrial ATP-synthase (complex V) may also be tractable targets. Since several members of each of these multi-subunit complexes show prognostic value, this provides an indication that inactivation, or specific modulation, of the activity of each of these complexes may provide significant therapeutic benefits. Therapeutic targeting of these complexes would be expected to prevent tumor recurrence and distant metastasis, as well as confer tamoxifen-sensitivity, in ER(+) breast cancer patients.

## MATERIALS AND METHODS

### Kaplan-Meier (K-M) analyses

To perform K-M analysis on > 400 nuclear mitochondrial gene transcripts, we used an open-access online survival analysis tool to interrogate publically available microarray data from up to 3,455 breast cancer patients [12]. This allowed us to determine their prognostic value. For this purpose, we primarily analyzed data from ER(+) patients that were LN(+) at diagnosis and were of the luminal A sub-type, that were primarily treated with tamoxifen and not other chemotherapy (*N* = 145 patients). In this group, 100% the patients received some form of hormonal therapy and ∼95% of them received tamoxifen. Biased and outlier array data were excluded from the analysis. This allowed us to identify > 60 nuclear mitochondrial gene transcripts, with significant prognostic value. Hazard-ratios were calculated, at the best auto-selected cut-off, and p-values were calculated using the logrank test and plotted in R. K-M curves were also generated online using the K-M-plotter (as high-resolution TIFF files), using univariate analysis:

http://kmplot.com/analysis/index.php?p = service&cancer = breast

This allowed us to directly perform *in silico* validation of these mitochondrial biomarker candidates. The multi-gene classifier function of the program was used to test the prognostic value of short mitochondrial gene signatures, using the mean expression of the selected probes. The 2012 version of the database was originally utilized for all these analyses, because a higher percentage of the patients used tamoxifen; however, virtually identical results were also obtained with the 2014 and 2017 versions.

## SUPPLEMENTARY FIGURES



## Abbreviations

CSCs: cancer stem-like cells
DMFS: distant metastasis-free survival
ER: estrogen receptor alpha (ESR1)
HR: hazard ratio
K-M: Kaplan-Meier
LN: lymph node
MRPL: mitochondrial ribosomal proteins, large subunit
MRPS: mitochondrial ribosomal proteins, small subunit
N: number of patients in a given data set
OXPHOS: oxidative phosphorylation (mitochondrial respiration)
RFS: recurrence- or relapse-free survival
