# Supplementary material for: Mitochondrial markers predict recurrence, metastasis and tamoxifen-resistance in breast cancer patients: Early detection of treatment failure with companion diagnostics
Source: Oncotarget. 2017 Jul 27;8(40):68730–45. doi: 10.18632/oncotarget.19612 (PMC5620292; doi:10.18632/oncotarget.19612)
Supplement: Supplementary file 1 [file oncotarget-08-68730-s001.pdf]

Mitochondrial markers predict recurrence, metastasis and tamoxifen-resistance in breast cancer patients: Early detection of treatment failure with companion diagnostics

SUPPLEMENTARY MATERIALS

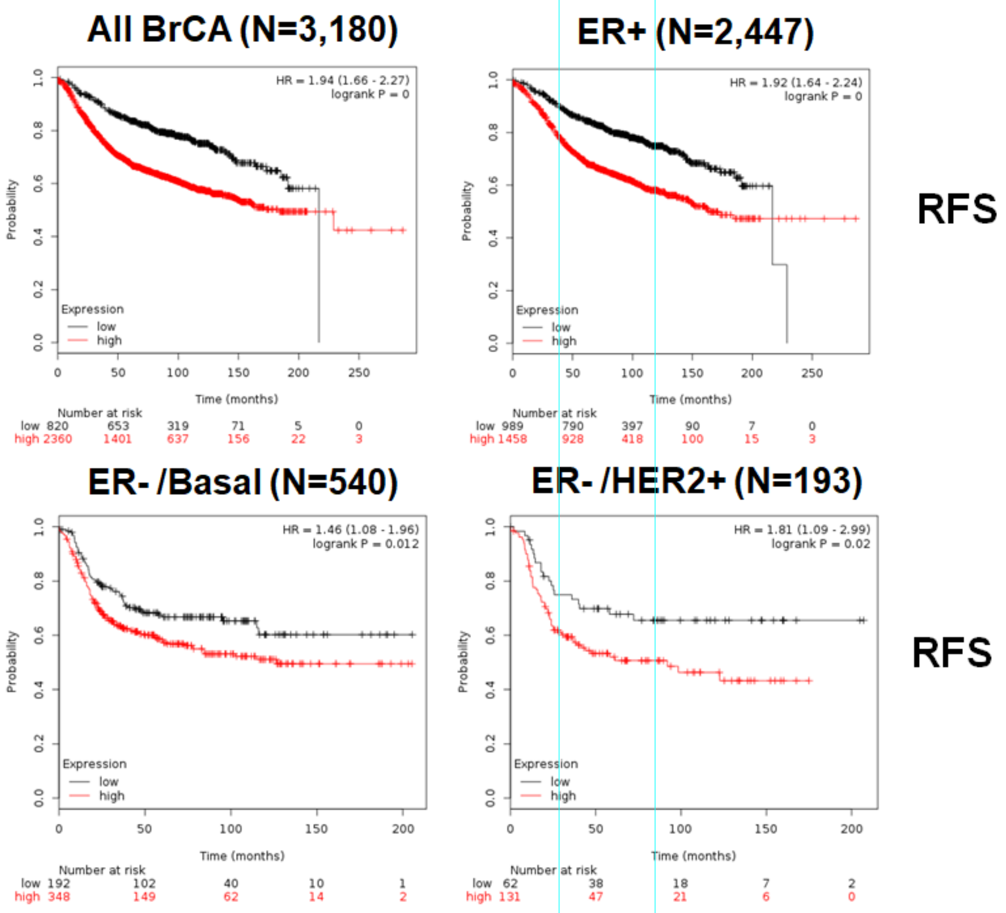

Supplementary Figure 1: Mito-Signature-1 still retains its predictive value in all breast cancers, ER(+) breast cancers, and ER(-) breast cancers (basal versus HER2(+)), in larger patient groups that were not segregated based on treatment. Patient numbers are as shown. P=0 indicates that the p-value is below 10-16. RFS, recurrence free survival (a.k.a., tumor recurrence).

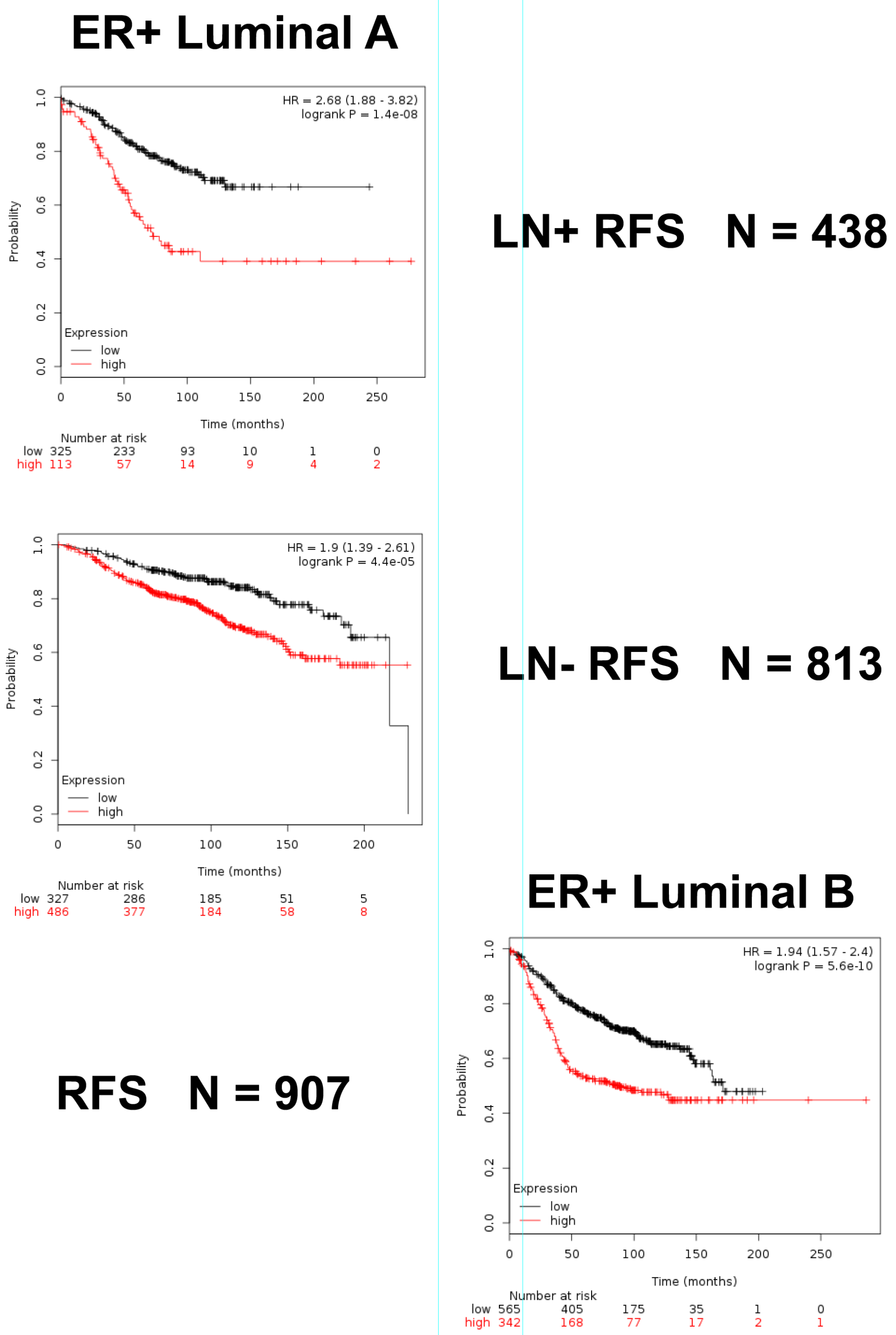

Supplementary Figure 2: Mito-Signature-1 still retains it predictive value in larger groups of luminal A (LN(+) versus LN(-)) and luminal B patients, that were not segregated based on treatment. Patient numbers are as shown. RFS, recurrence free survival (a.k.a., tumor recurrence).
